# Site-specific apparent optimum air temperature for vegetation photosynthesis across the globe

**DOI:** 10.1038/s41597-024-03603-7

**Published:** 2024-07-11

**Authors:** Li Pan, Xiangming Xiao, Yuan Yao, Baihong Pan, Chenglong Yin, Cheng Meng, Yuanwei Qin, Chenchen Zhang

**Affiliations:** School of Biological Sciences, Center for Earth Observation and Modeling, University of Oklahoma, Norman, OK 73019 USA

**Keywords:** Ecological modelling, Natural variation in plants, Ecological modelling

## Abstract

The apparent optimum air temperature for vegetation photosynthesis (T_opt_) is a key temperature parameter in terrestrial ecosystem models estimating daily photosynthesis or gross primary production (GPP, g C/m^2^/day). To date, most models use biome-specific T_opt_ (T_opt-biome_) parameter values. Given vegetation acclimation and adaptation to local climate, site-specific T_opt_ (T_opt-site_) is needed to reduce uncertainties in estimating daily GPP across the scales from site to region and the globe. Previous studies have demonstrated using the Enhanced Vegetation Index (EVI) derived from Moderate Resolution Imaging Spectroradiometer (MODIS) images and daytime air temperature data to estimate the T_opt-site_ at the eddy covariance tower sites. This study used MODIS-derived EVI and ERA5 climate data to estimate and generate global T_opt-site_ data products from 2000 to 2019. The T_opt-site_ of individual pixels within a biome has large variation, which clearly cannot be represented accurately by the widely used T_opt-biome_. Therefore, using this global dataset of T_opt-site_ estimates might significantly affect GPP simulation in current ecosystem models.

## Background & Summary

Among the carbon flux components, Gross Primary Production (GPP) represents the amount of carbon dioxide absorbed by vegetation, playing a pivotal role in the global carbon cycle^1,2^. Air temperature (T_air_, ~2 m above the ground) is one of the key climatic variables influencing the spatiotemporal dynamics of GPP^2–4^. An increase in T_air_ can boost enzyme activity, leading to a rise in photosynthesis rate, and excessive T_air_ can result in stomatal closure and enzyme deactivation, causing a decline in photosynthesis^5,6^. Therefore, the GPP response to T_air_ follows a parabolic (unimodal) curve^4,7^, and the apparent optimum air temperature (T_opt_) is generally defined as the T_air_ at which vegetation achieves its peak photosynthesis rate.

Almost all terrestrial ecosystem carbon cycle models have temperature response functions for GPP, with T_opt_ being a crucial temperature parameter^8,9^. Light Use Efficiency (LUE) models also have the T_opt_ parameter in estimating GPP^10–12^. Currently, the T_opt_ parameter values in terrestrial ecosystem models use biome-specific T_opt_ for individual biomes (T_opt-biome_)^13,14^. However, recent studies suggest that the T_opt-biome_ may significantly differ from site-specific T_opt_ (T_opt-site_)^15,16^. T_opt-biome_ parameter values used in the models came from analyses of the limited number of locations for individual biomes^16^. Additionally, because of vegetation acclimation and adaption to local climate^8,17^, the use of biome-specific T_opt_ at the global scale could introduce large errors and uncertainty in global GPP estimation.

GPP and air temperature data from eddy covariance (EC) flux tower sites are widely used to study the response curves of GPP to air temperature and to estimate the T_opt-site_ at individual sites (ecosystems)^4,8,16,18–20^. However, the number of EC towers is limited^21^, and their footprints only cover a small portion of the global land surface^22^. Additionally, most EC flux tower observations last a few years or a decade, making it challenging to explore the interannual variations of T_opt_ at the sites_._ Several studies explored using remote sensing data to estimate T_opt-site_ over large geographical domains and longer periods^15,18,23^. Since the photosynthesis rate depends on the number of photosynthetic organs (i.e., chloroplasts) in the canopy, it can be directly represented by the total chlorophyll content in the canopy^24,25^. Many studies have explored the estimation of canopy chlorophyll content from satellite-derived vegetation indices (VIs), such as the Enhanced Vegetation Index (EVI)^26,27^, Normalized Difference Vegetation Index (NDVI)^28^, and Near-infrared Reflectance of Vegetation (NIRv)^29^. These VIs have shown a strong relationship with GPP and are therefore used as predictor variables to represent vegetation structure and function^23,25,30,31^. Recent studies have demonstrated the feasibility of using VIs-T response curves to estimate T_opt-site_ at site, regional, or global scales^7,15,20,23,32^. It is worth noting that these publications differ in terms of VIs variables (e.g., NDVI, EVI, and NIRv), air temperature datasets (e.g., daily mean air temperature), and algorithms, leading to noticeable discrepancies in T_opt-site_ estimates across these studies. Accurate quantification of T_opt-site_ and site-year-specific T_opt_ (T_opti-site-year_) at global scale remains challenging.

In this study, built upon our previous studies that used GPP, EVI, and daytime air temperature data to estimate T_opt-site_ at the eddy covariance tower sites^7,20^, we first generated the T_opt-site-year_ and T_opt-site_ dataset across the globe from 2000 to 2019, using MODIS-derived EVI data (500-m spatial resolution) and daytime air temperature from ERA5 climate data^33,34^. We also carried out exploratory data analyses of this dataset, which provides basic information on (1) the spatiotemporal patterns of T_opt-site-year_ and T_opt-site_ across the globe over the past two decades and (2) the variability (frequency distribution) of T_opt-site_ from all the pixels within individual biomes. Terrestrial ecosystem models could use this global T_opt-site_ dataset to increase the accuracy of and reduce the uncertainty of GPP data under historical, current, and future climate scenarios. The T_opt-site_ dataset could also be used for further analyses that offer valuable insights into how vegetation has responded to climate change over the past two decades.

## Methods

### Datasets and pre-processing

#### ERA5-Land reanalysis climate data

ERA5 is the fifth generation of atmospheric reanalysis products released by the European Centre for Medium-Range Weather Forecasts (ECMWF)^33^. This dataset offers comprehensive historical and near-real-time information on global atmospheric, terrestrial, and oceanic variables^34^. ERA5-Land complements the ERA5 by focusing specifically on terrestrial processes. Compared to ERA5, ERA5-Land boasts a higher spatial resolution (0.1°) and a higher temporal resolution (hourly), enabling a more precise depiction of surface processes and characteristics, including air temperature and precipitation.

In this study, we used the air temperature (T_air_) layer from the ERA5-Land reanalysis climate dataset. We provided an example of the air temperature data from the Harvard Forest site (US-Ha1) in 2010 (Fig. 2) and illustrate the discrepancies among the three temperature variables: daily maximum air temperature (T_air-max_), daily daytime mean air temperature (T_air-DT_), and daily mean air temperature (T_air-mean_); and we assessed the appropriateness of these three temperature variables for the T_opt_ estimation algorithm. T_air-DT_ is defined by averaging T_air-max_ with T_air-mean_^10^:

1 $${{\rm{T}}}_{{\rm{air}}{\rm{ \mbox{-} }}{\rm{DT}}}={\rm{average}}({{\rm{T}}}_{{\rm{air}} \mbox{-} \max },{{\rm{T}}}_{{\rm{air}} \mbox{-} {\rm{mean}}})$$

We aggregated these three air temperature variables from daily data into 8-day intervals by averaging the observations over the 8-day period to align with the temporal resolution of MOD09A1.

#### MODIS land surface temperature dataset

**Fig. 1:**
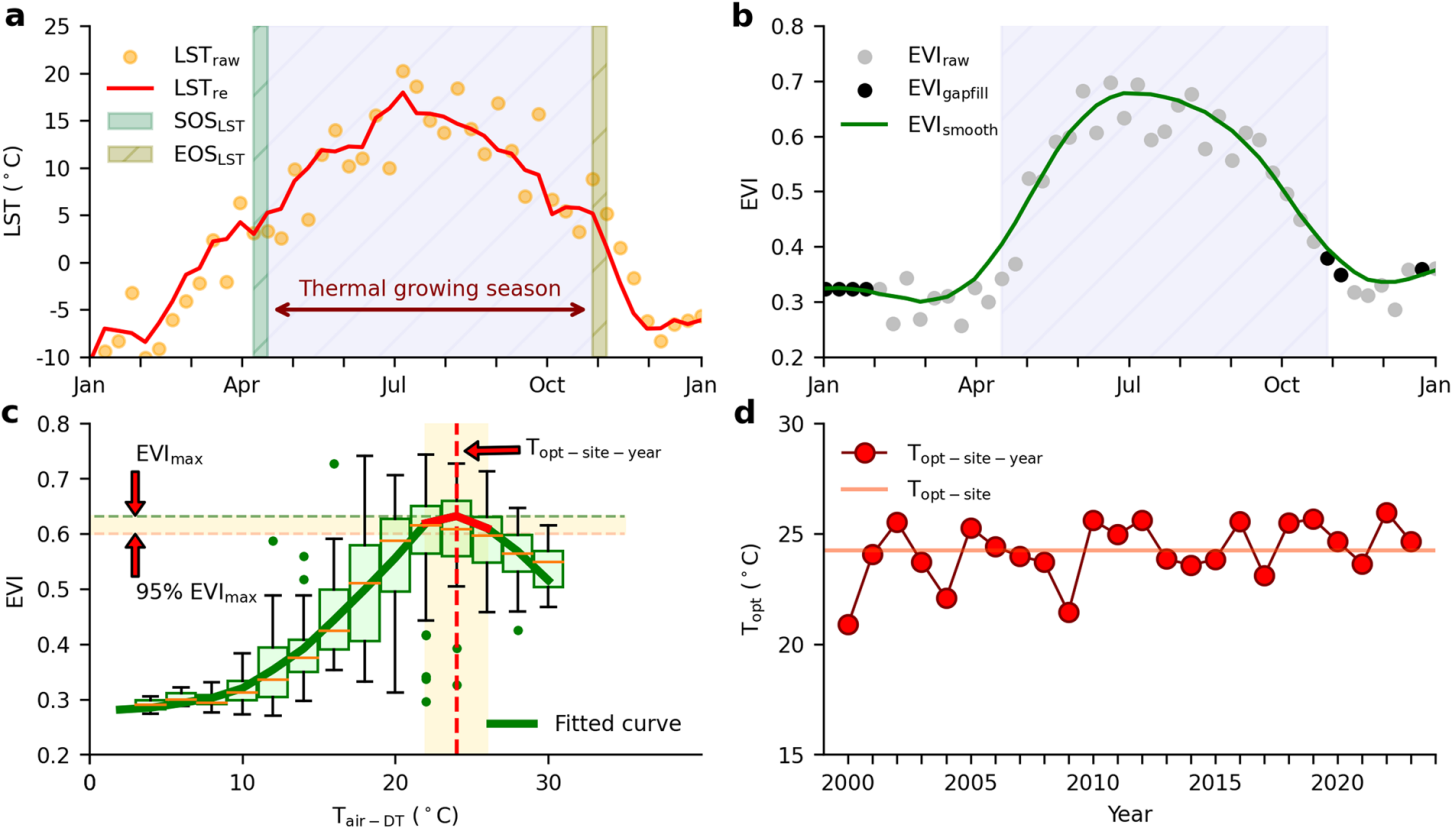
The workflow for estimating site-specific apparent optimum temperature (T_opt-site_) for vegetation photosynthesis at a deciduous broadleaf forest site in the USA (Harvard Forest, US-Ha1, DBF, 42.5378°N, 72.1715°W). The US-Ha1 site was selected because it is a mature forest subject to long-term observation and research, showing the typical seasonal dynamics of vegetation in mid-latitude areas. The altitude is 340 meters. In contrast, we also selected the Amazon forest site BR-Sa3 to show the seasonal dynamics of vegetation in tropical regions (Fig. S1). (**a**) raw LST (LST_raw_) and reconstructed LST (LST_re_) time series. The thermal growing season is defined as the period LST_re_ greater than 5 °C (light blue filled area). SOS_LST_ and EOS_LST_ represent the start and end of the thermal growing season, respectively; (**b**) raw EVI (EVI_raw_, only after cloud removal), gap-filled EVI (EVI_gapfill_, linear interpolation), and smoothed EVI (EVI_smooth_, S-G filter); (**c**) The curve of EVI-T_air_. The solid green curve is fitting curves using cubic regression splines. (**c**) also shows a boxplot with points every 2 °C bin; (**d**) interannual variations of T_opt-site-year_ from 2000 to 2019, T_opt-site_ is calculated as the median value from 20 years of T_opt-site-year_ data.

The Moderate Resolution Imaging Spectroradiometer (MODIS) is a key instrument for observing changes on the Earth’s surface^35^. The MYD11A2 dataset provides an 8-day and 1-km composite of global land surface temperatures (LST) and emissivity, sourced from the MODIS instrument aboard NASA’s Aqua satellite. This dataset provides LST at 1:30 a.m. and 1:30 p.m. In this study, we constrained the analyses of EVI and T_air_ data and T_opt_ algorithm within the thermal growing season^36^, as defined by nighttime LST^37^, which helps to mitigate uncertainties from outliers beyond this period (e.g., high EVI caused by snow). For each pixel (1-km spatial resolution), we reconstructed the LST time series using a moving window of three consecutive 8-day periods, calculating the average across them (Fig. 1a). Adopting the threshold assumption that the LST for the thermal growing season is > = 5 °C^38^, the start of the season (SOS_LST_) was marked by the LST first surpassing this threshold in winter/spring, while its end of the season (EOS_LST_) corresponds to the LST first descending below this threshold in fall/winter (Fig. 1a). Note this method was applied only to those pixels where the annual peak LST surpasses 5 °C. For areas with an annual peak LST not reaching 5 °C (e.g., high-altitude plateau or boreal forests), we substituted LST with T_air-DT_.

#### MODIS surface reflectance and vegetation indices

The MODIS surface reflectance products are widely used in monitoring global vegetation^39^. In this study, we utilized the MOD09A1 Surface Reflectance V6.1 product, a dataset of seven spectral bands ranging from 0.459μm to 2.155μm with a 500-m spatial and an 8-day temporal resolution^40^.

We have processed and analyzed the global MOD09A1 surface reflectance time series data from 2000 to 2019. Three vegetation indices - NDVI, EVI, and NIRv - were calculated. NDVI, utilizing near-infrared and red bands, offers a measure of vegetation greenness and is hence used to identify green vegetation areas^41^. EVI, while similar to NDVI, is specifically designed to correct for atmospheric disturbances and background soil effects^42^. NIRv is derived from NDVI, yet it exhibits a stronger correlation with observed GPP than NDVI^43^. Among these vegetation indices, EVI has been reported to be most closely related to leaf and canopy-level chlorophyll content^26–28,31,44^. Additionally, several biophysical studies have shown that EVI is nearly unsaturated and sensitive to canopy changes^26,31^. The three VIs are calculated by the formulas (2–4):

2 $${\rm{NDVI}}=\frac{{{\rm{\rho }}}_{{\rm{nir}}}-{{\rm{\rho }}}_{{\rm{red}}}}{{{\rm{\rho }}}_{{\rm{nir}}}+{{\rm{\rho }}}_{{\rm{red}}}}$$

3 $${\rm{EVI}}=2.5\times \frac{{{\rm{\rho }}}_{{\rm{nir}}}-{{\rm{\rho }}}_{{\rm{red}}}}{{{\rm{\rho }}}_{{\rm{nir}}}+(6\times {{\rm{\rho }}}_{{\rm{red}}}-7.5\times {{\rm{\rho }}}_{{\rm{blue}}})+1}$$

4 $${\rm{NIRv}}={\rm{NDVI}}\times {{\rm{\rho }}}_{{\rm{nir}}}$$

where ρ_nir_, ρ_red_, and ρ_blue_ are the surface reflectance bands of near infrared, red, and blue, respectively. In order to generate a high-quality and continuous surface reflectance time series, the QA-band quality detection approach was employed to retrieve pixels affected by clouds and cloud shadows. Additionally, the Best Index Slope Extraction (BISE) method was used to detect overlooked low-quality observation pixels^45^. After two rounds of filtration processes, the low-quality observations were then marked as a mask for the row image (Fig. 1b). A linear interpolation approach, coupled with high-quality observations, was used to fill the gaps^46,47^ (Fig. 1b). Finally, to further minimize the temporal variation within the time series data, we utilized the Savitzky-Golay (S-G) method for curve smoothing^48^. Note that the smoothing window is set based on the varying lengths of the thermal growing season; for instance, if the thermal growing season of a pixel is 240 days, a sliding window of 31 8-day observations was employed for curve smoothing.

#### MODIS land cover product

The MODIS Land Cover Type (MCD12Q1) Version 6.1 data product offers annual global land cover classifications from 2001 to the present^49^. In this study, we utilized the International Geosphere Biosphere Program (IGBP) layer, which identifies 17 distinct land cover types. Our focus was on the following 14 categories: Evergreen Needleleaf Forests (ENF), Evergreen Broadleaf Forests (EBF), Deciduous Needleleaf Forests (DNF), Deciduous Broadleaf Forests (DBF), Mixed Forests (MF), Closed Shrublands (CSH), Open Shrublands (OSH), Woody Savannas (WSA), Savannas (SAV), Grasslands (GRA), Permanent Wetlands (WET), Croplands (CRO), Urban and Built-up Lands (URB), and Cropland/Natural Vegetation Mosaics (CNV). The spatial delineation of these categories is illustrated in Fig. S2. In this paper, we explored the variation of T_opt-site_ within individual biomes for the above-mentioned 14 biomes.

### Methods to estimate T_opt-site-year_ and T_opt-site_

#### Air temperature variables input

**Fig. 2:**
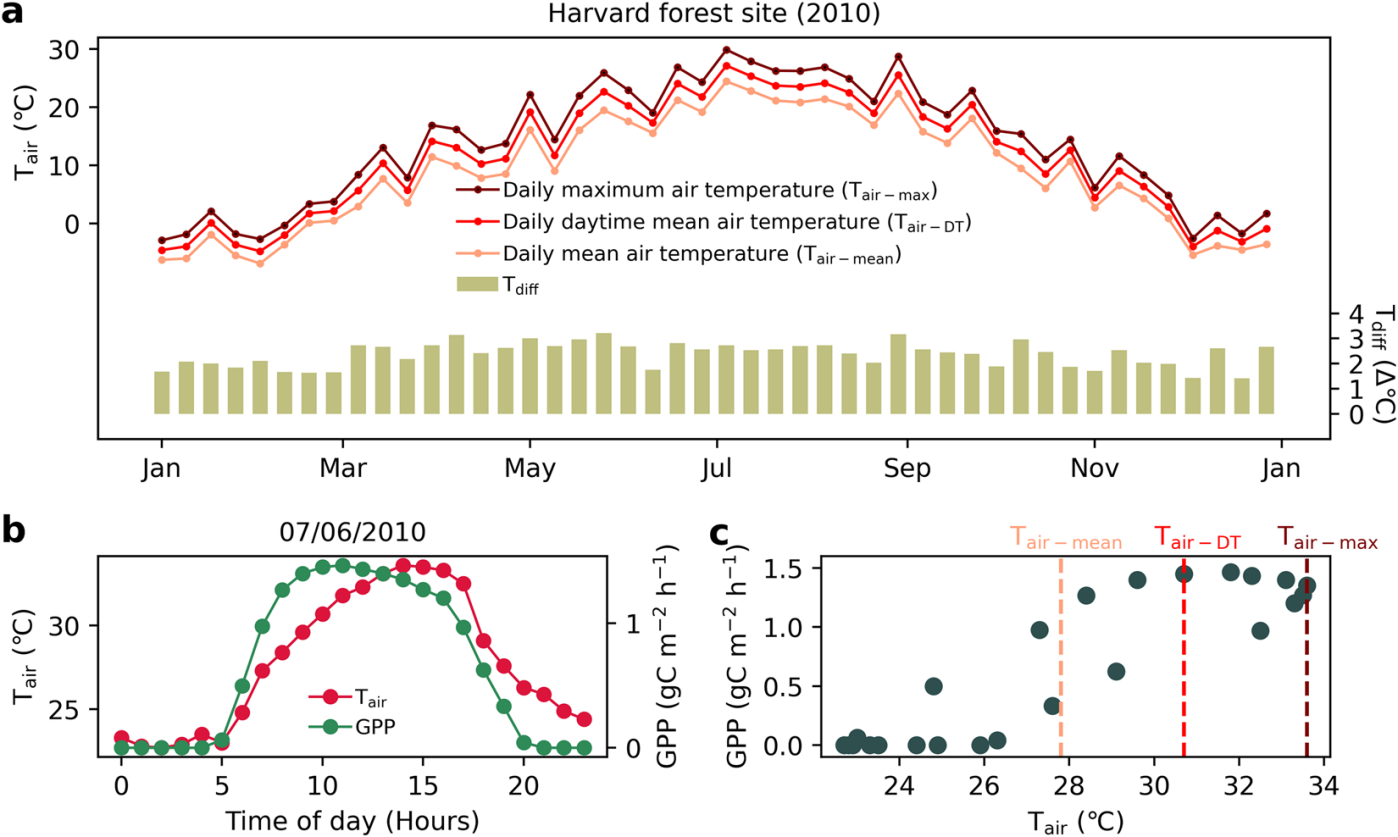
Temporal dynamics of air temperature and GPP_EC_ at the Harvard Forest eddy flux tower site (US-Ha1) in 2010. (**a**) Seasonal dynamics of daily maximum air temperature (T_air-max_), daily daytime mean air temperature (T_air-DT_), and daily mean air temperature (T_air-mean_). This figure also shows the difference between T_air-max_ and T_air-DT_ (T_diff_); (**b**) diurnal dynamics of GPP and T_air_; (**c**) 2-d scatter plot of GPP and T_air_.

We investigated the seasonal dynamics of T_air-max_, T_air-DT_, and T_air-mean_ within one year (Fig. 2a, the case at Harvard Forest site). The discrepancy between T_air-max_ and T_air-DT_ (or between T_air-DT_ and T_air-mean_) can be as high as 3 °C throughout the whole year.

A further examination of the diurnal dynamics of GPP and T_air_ is presented in Fig. 2b. Interestingly, the diurnal peaks of GPP and T_air_ were not consistent. GPP reached its peak between 9:00 a.m. and 11:00 a.m., whereas T_air_ peaked later, from 2:00 p.m. to 4:00 p.m. Of particular note is that among the three temperature variables, T_air-DT_ most closely approximated the temperature of T_air_ at the moment GPP peaked (Fig. 2c). Given these observations, T_air-DT_ is regarded as the most appropriate air temperature variable for the algorithm input.

#### Algorithm of T_opt-site-year_ and T_opt-site_

T_opt-site-year_ is derived from the annual response curve of EVI to T_air-DT_^16,20^. For each year, we plotted the response curve of EVI versus T_air-DT_ with all observations within the thermal growing season (Fig. 1c). Then, the curve fitting of EVI against T_air-DT_ was performed using cubic regression splines. The optimum T_air-DT_ range for vegetation photosynthesis is defined as when EVI exceeds 95% of its maximum value, and T_opt-site-year_ is determined from the average of all T_air-DT_ within that range (Fig. 1c). T_opt-site_ was calculated as the median value of T_opt-site-year_ across all 20 years (Fig. 1d).

## Data Records

The datasets include T_opt-site-year_ with 500-meter spatial resolution across the globe from 2000 to 2019, labeled as “Global_Topt_site_year_“ followed by the specific year, and T_opt-site_, labeled as “Global_Topt_site”. The units are in °C with a scalar factor of 0.01. The maps only show the areas where the annual mean NDVI is greater than 0.1 (vegetated pixel). The datasets have been submitted to the *figshare* data repository portal: 10.6084/m9.figshare.24514459.v6^50^.

**Fig. 3:**
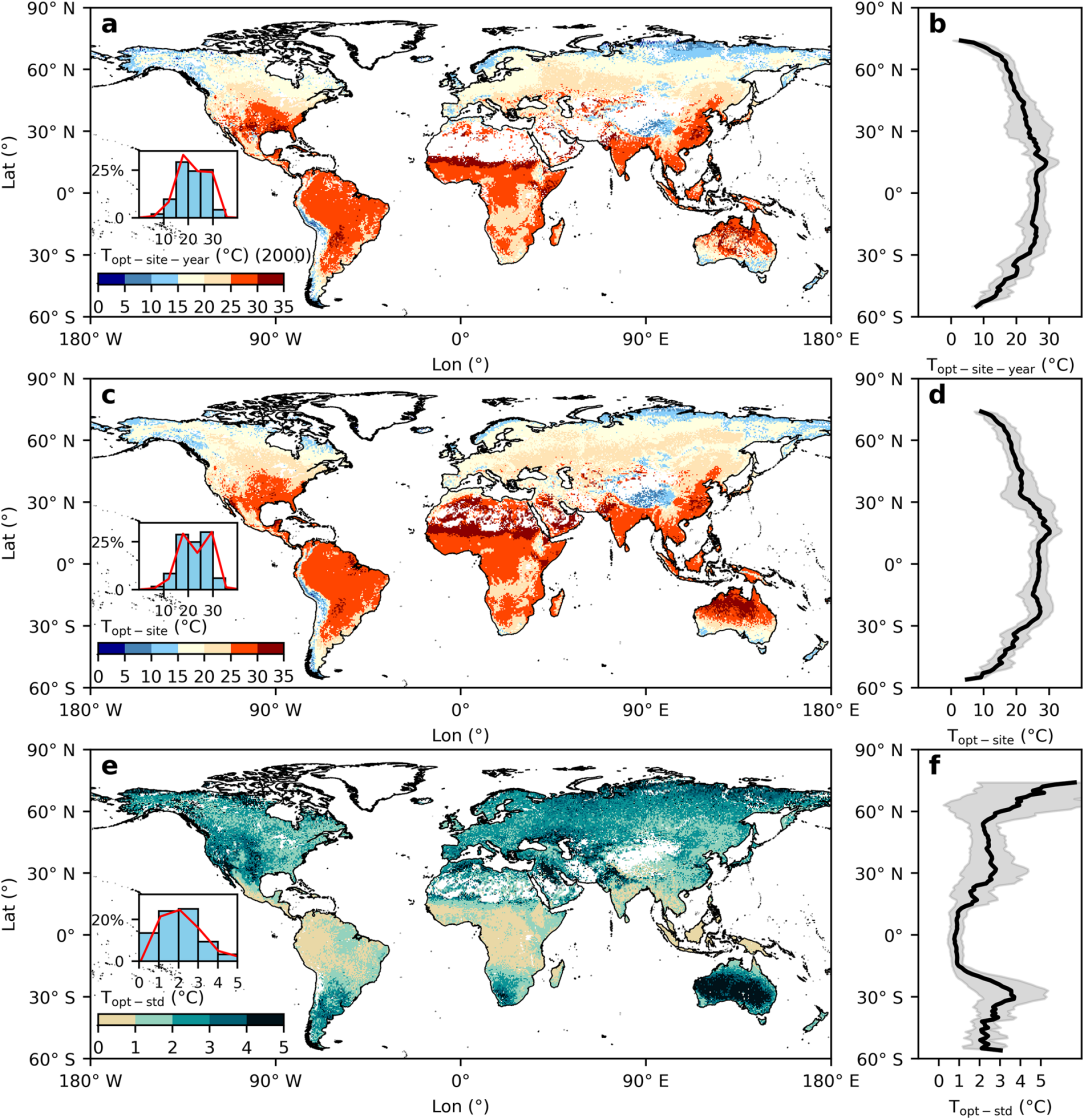
Global maps of apparent optimum air temperature and interannual variation. (**a**) global map of T_opt-site-year_ in 2000; (**c**) global map of T_opt-site_, derived from the median value of T_opt-site-year_ from 2000 to 2019; (**e**) T_opt-std_ is calculated as the standard deviation of T_opt-site-year_ during the period. The global map only shows the area where the annual NDVI is larger than 0.1 (The blank areas are pixels where the annual NDVI is less than 0.1). The histogram represents the proportion of pixels within the different intervals, as shown on the x-axis. The red line represents the Kernel Density Estimation (KDE). (**b,c,f**) correspond to the average values across the latitude gradient in (**a,c,e**), respectively. The solid black lines are calculated as the average value for all pixels within 1° of latitude, and the shadow indicates the standard deviation.

Fig. 3a shows the global distribution of T_opt-site-year_ for the year 2000. From a global perspective, 91% of the vegetation has their T_opt-site-year_ within the 10 to 30 °C range, while 3% and 6% have their T_opt-site-year_ below 10 °C and above 30 °C, respectively (Fig. 3a). Across the latitudinal gradient, T_opt-site-year_ ranges between 4 to 35 °C (Fig. 3b). The highest T_opt-site-year_ is observed in the tropics. The T_opt-site-year_ value declines with a northward or southward progression in latitude, decreasing by an average of 0.34 °C for each 1° higher latitude, reaching its minimum near the poles. In high-altitude regions, such as the Qinghai-Tibet Plateau and Andes Mountains, the T_opt-site-year_ is significantly low.

Fig. 3c shows the global map of T_opt-site_, derived from the median value of two decades of T_opt-site-year_. The spatial distribution and its trend across the latitude gradient mirror the patterns observed for T_opt-site-year_ in 2000 (Fig. 3c,d). Comparing the histograms in Fig. 3c and Fig. 3a, the T_opt-site_ shows a slight increase relative to the T_opt-site-year_ from 2000. Notably, between 25 °C and 30 °C, the proportion of vegetation with this T_opt-site_ has marginally increased (~approximately 5% pixels higher). We further investigate the trends at five-year intervals (Fig. S3). The T_opt-site-year_ exhibited a slight global increasing trend, predominantly observed in arid regions such as central-northern Australia, North Africa, and southern North America. In these areas, the T_opt-site-year_ showed an increase exceeding 5 °C.

Fig. 3e presents the standard deviation of the global T_opt-site-year_ (T_opt-std_) over the last two decades. Between 2000 and 2019, the histogram shows that T_opt-std_ primarily fluctuated between 1 °C and 3 °C for 71% of the vegetation pixels in the globe. Meanwhile, 14% of the vegetation exhibited relative stability with interannual variations of less than 1 °C, and 15% of vegetation experienced fluctuations greater than 3 °C. Across the latitude gradient (Fig. 3f), the largest T_opt-std_ predominantly occurs in the northern boreal and southern temperate zones. Within the northern boreal zone, the T_opt-std_ increases with latitude, up to 3 °C. In the southern temperate zone, Australia manifests the largest T_opt-std_, up to 5 °C. Tropical regions exhibit the smallest T_opt-std_ with an interannual variability of around 1 °C, followed by the northern temperate zone, which shows a moderate fluctuation ranging from 2 to 3 °C.

**Fig. 4:**
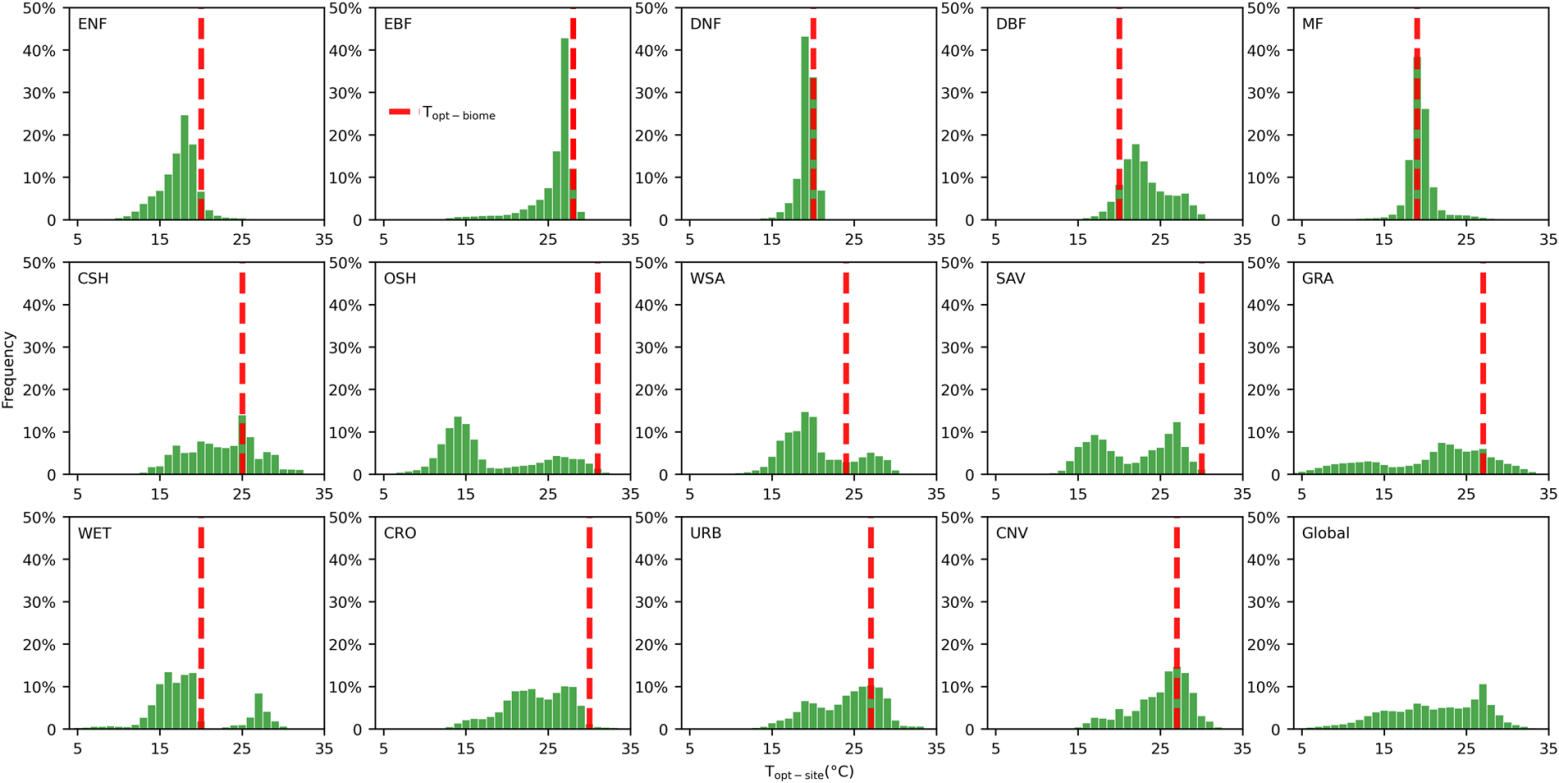
Histogram of T_opt-site_ within 14 biomes. The red line is T_opt-biome_, a common parameter estimated by Raich *et al*.^13^ and Melillo *et al*.^14^, which has been widely used in most ecological models^13,14^.

The histogram in Fig. 4 shows the range (frequency distribution) of T_opt-site_ across 14 biomes, revealing significant variation of T_opt-site_ due to geographical and climatic differences. For instance, the GRA exhibits a wide T_opt-site_ range from 4 °C to 35 °C, reflecting their extensive global distribution. In contrast, the DNF, primarily found in colder boreal zones, has a narrower range of 14 °C to 21 °C.

## Technical Validation

### Comparison against T_opt-site_ derived from eddy covariance flux tower (${{\rm{T}}}_{{\rm{opt}}-{\rm{site}}}^{{\rm{GPP}}}$)

**Fig. 5:**
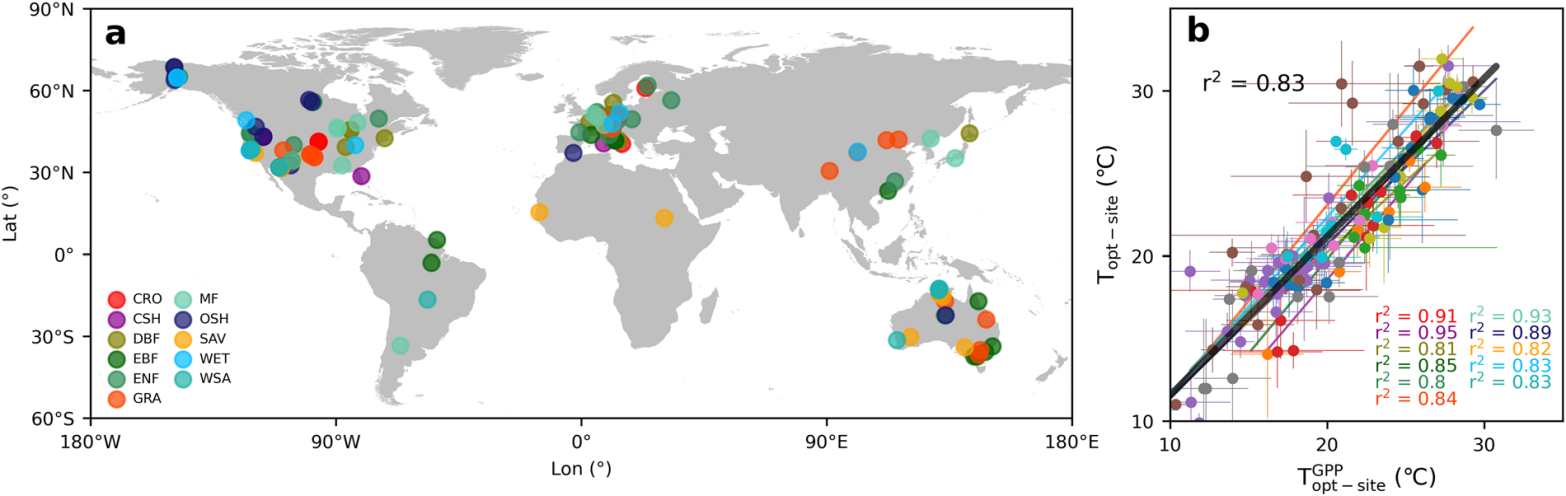
The comparison with T_opt-site_ and ${T}_{\mathrm{opt} \mbox{-} \mathrm{site}}^{\mathrm{GPP}}$ across the globe. (**a**) The 137 eddy flux tower sites across the 11 biomes in the globe; (**b**) Relationship between T_opt-site_ and ${{\rm{T}}}_{{\rm{opt}} \mbox{-} {\rm{site}}}^{{\rm{GPP}}}$. Error bars indicate ± S.D. from T_opt-site-year_ and ${{\rm{T}}}_{{\rm{opt}} \mbox{-} {\rm{site}} \mbox{-} {\rm{year}}}^{{\rm{GPP}}}$, respectively. Different colors indicate different biomes, and the black solid line indicates all biomes.

We compared the T_opt-site_ (derived from EVI) with the ${{\rm{T}}}_{\mathrm{opt} \mbox{-} \mathrm{site}}^{\mathrm{GPP}}$ (derived from GPP, see Supplementary methods 1) at 137 eddy flux tower sites across 11 out of 14 biomes (DNF, URB, and CNV were excluded due to their very limited number of sites) in the globe (Fig. 5a). We employed a simple linear regression analysis (see Supplementary methods 2) to investigate the relationship between T_opt-site_ and ${T}_{\mathrm{opt} \mbox{-} \mathrm{site}}^{\mathrm{GPP}}$. The T_opt-site_ generated in this study showed high consistency with ${T}_{\mathrm{opt} \mbox{-} \mathrm{site}}^{\mathrm{GPP}}$ across all 11 biomes (r^2^ between 0.80 and 0.95) (Fig. 5b). The black solid line represents the overall linear fit for all biomes, exhibiting an r² of 0.83 (By comparison, the number is 0.52 in Huang *et al*.^15^ across 125 sites), indicating that the T_opt-site_ estimated in this study is reliable at the ecosystem scale.

### Comparison of materials and methods used to estimate T_opt-site_

We compiled previous studies that utilized GPP/NEE/VIs-T_air_ response curves for estimating T_opt-site_ (Table 1). These studies varied in terms of VIs variables, air temperature variables, and algorithms, leading to noticeable discrepancies in global T_opt-site_ estimations. For instance, two studies estimated the mean global T_opt_ at 18.8 ± 7.1 °C^18^ and 23 ± 7.8 °C^15^, respectively. In contrast, this study estimates it to be 22 ± 6 °C. The discrepancies highlight the necessity and challenges associated with reducing uncertainties in variables, data sources, and algorithms.

**Table 1:** Comparison of variables (vegetation indices, climate), data source, study area, algorithms, and output in this and previous studies.

Studies	Variables	Data source	Study area	Algorithms	Output
This study	EVI vs. T_air-DT_	MOD09A1; ERA5-Land	Global	T_opt_ is defined as the T_air-DT_ at which EVIs exceed 95% of EVI_max_.	T_opt-site-year_, T_opt-site_
Chang *et al*.^20^; Chang *et al*.^7^	EVI vs. T_air-DT_ GPP vs. T_air-DT_	MOD09A1, Eddy flux tower	165 sites, 11 grassland sites	T_opt_ is defined as the T_air-DT_ at which EVIs exceed 95% of EVI_max_.	T_opt-site-year_, T_opt-site_
Huang *et al*.^15^; Chen *et al*.^32^	NIRv vs. T_air-max_	Climatic Research Unit/National Centers for Environmental Protection (CRU/NCEP)	Global, Tibetan Plateau	T_opt_ is defined as the T_air-max_ at which the maximum of NIRv occurs	T_opt-site_
Potter *et al*.^51^	NDVI vs. T_air-mean_	Monthly NDVI-FASIR; Historical climate data	Global	T_opt_ is defined as the monthly T_air-mean_ corresponding to the month when the maximum NDVI occurs	T_opt-site_
Cui *et al*.^52^	NDVI vs. T_air-mean_	GIMMS NDVI; 752 meteorological stations over China	China	T_opt_ is defined as the average of the T_air-mean_ corresponding to the peak NDVI values and the T_air-mean_ at which NDVI exhibits its maximum rate of increase	T_opt-site_
Niu *et al*.^8^; Wang *et al*.^18^	GPP vs.T_air-mean_	Eddy flux tower	169 sites, 326 sites, global	T_opt_ is defined as the T_air-mean_ at which the maximum of GPP occurs	T_opt-site-year_, T_opt-site_
Yuan *et al*.^19^	NEE vs. T_air-mean_	Eddy flux tower	72 sites	T_opt_ is defined as the T_air-mean_ at which maximized carbon uptake is occurs	T_opt-site_
Bennett *et al*.^4^	instantaneous GPP vs. T_air_	Eddy flux tower	17 wooded ecosystem sites in Australia	T_opt_ is defined as the instantaneous T_air_ at which maximized instantaneous GPP occurs	T_opt-site_

In terms of vegetation index variables used in estimating T_opt-site_, NDVI, as one of the earliest and most commonly used vegetation indices^51,52^, might introduce biases when estimating T_opt-site_ based on the NDVI- temperature response curve. This bias is attributed to the characteristics of NDVI dynamics in the plant growing season. In those sites of dense forest with high leaf area index (> = 4 m^2^/m^2^), NDVI may saturate and remain high throughout the summer, which leads to a shift in the T_opt_ window from early summer to mid-summer, causing an overestimation of T_opt_ (Fig. S4). In the later summer, although NDVI remains high, GPP has already begun to decline^23^, The histograms of NDVI-derived T_opt-site_ map show that pixels with T_opt-site_ over 20 °C make up 64% (Fig. S5a), whereas this proportion is 55% in EVI-derived T_opt-site_ map (Fig. 3b). Recent studies employed NIRv to generate regional and global T_opt-site_ maps^15,32^. Given that EVI exhibits a higher correlation with GPP than NDVI in most ecosystems like forests^26,27^, crops^53,54^, and grasslands^55^, it can be regarded as an ideal proxy to study vegetation response to temperature variations. Our findings also show that the T_opt-site_ map derived from NIRv has similar global patterns and magnitudes with those derived from EVI (Fig. S5c). This consistency can be attributed to the close correlation of EVI and NIRv, and both of them also have strong correlation with solar-induced chlorophyll fluorescence (SIF), which is the amount of light emitted by plants^15,43^.

In terms of air temperature variables used in estimating T_opt-site_, most studies employ two types of air temperature variables: T_air-max_^15,32^ and T_air-mean_^8,18,19^. However, the peak of vegetation photosynthesis does not occur at the hottest moment of the day (as shown in Fig. 2). The maximum GPP typically occurs in the later morning, while T_air-max_ tends to occur in the earlier afternoon. This renders T_air-max_ an inappropriate choice for air temperature variables in the analyses of air temperature and GPP relationship, as it tends to overestimate T_opt-site_ at the higher end, i.e., a bias of higher T_opt-site_ values. Since photosynthesis only happens during daytime and T_air-mean_ factors in nighttime temperatures, the use of T_air-mean_ will introduce a bias of lower T_opt-site_ values. Previous studies have already demonstrated the potential and feasibility of estimating T_opt_ based on the EVI-T_air-DT_ response curve at a limited number of sites^7,20^, and to our knowledge, this study is the first time that T_air-DT_ was used as variable inputs to estimate global T_opt-site_.

In terms of algorithms, most studies defined T_opt_ as the air temperature corresponding to the highest point of the VI-T_air_ or GPP-T_air_ response curve with multi-year data^4,8,15,18,19,32^; for example, the recent global T_opt-site_ estimates by Huang ey al.^15^. Different with those studies, this study first calculated annual T_opt-site-year_ and then estimated T_opt-site_ as the median of T_opt-site-year_ in 20 years. The advantage of this method is that T_opt-site-year_ provides the variation information of T_opt-site_ across years, especially considering the substantial inter-annual variations in climate and land cover in the past two decades in the globe. T_opt-site-year_ data would help expand the understanding of how vegetation responds to environmental changes over time. In addition, abnormal vegetation growth caused by extreme climate events in specific years may also introduce uncertainty to the estimation. We use the median value of T_opt-site-year_ over multiple years, as the median value is primarily used to eliminate potential outliers and will provide a more balanced perspective of the T_opt-site_ over time. Additionally, we improved the T_opt-site_ estimation window based on the thermal growing season defined by LST, which significantly reduced the uncertainty introduced by snow in the algorithm, particularly in high-latitude regions.

### Spatial variation of T_opt-site_ across biomes from this study and its comparison with T_opt-biome_

The range of T_opt-site_ highlights the limitations of using a fixed T_opt-biome_ parameter. T_opt-biome_ shows a substantial discrepancy when compared with the T_opt-site_ across biomes. This discrepancy is especially pronounced in such biomes as ENF, OSH, SAV, and CRO, where the T_opt-site_ is always lower than the T_opt-biome_. This bias is mainly due to the T_opt-biome_ being based on limited site data, further introducing significant uncertainty into the GPP estimation^7,20,56,57^. Our previous research suggests that replacing the T_opt-biome_ with T_opt-site_ can significantly improve the accuracy of GPP estimates from the model at site scale by approximately 1% to 34%, especially in the GRA, CRO, and OSH ecosystems^16^. This highlights the importance of using the T_opt-site_ rather than the T_opt-biome_ for simulations of GPP models across the scales from site to region and the globe.

## Usage Notes

The T_opt-site_ dataset produced in this study can be used as an input for most ecosystem models that include temperature parameters. Note that we have masked areas with an annual average NDVI of less than 0.1 (such as deserts, glaciers, or areas with high-density impervious surfaces). Furthermore, T_opt-site_ is highly related to plant physiology, ecology, and climatology. The T_opt-site-year_ dataset could be used to explore the significant importance of understanding plant growth’s environmental dependence, ecosystems’ functioning, and their responses to environmental changes.

### Supplementary information

Supplementary Online materials for “Site-specific apparent optimum air temperature for vegetation photosynthesis across the globe”

## Data Availability

The algorithm is run on Google Earth Engine (GEE) and the code can be found at this link: 10.6084/m9.figshare.24587259^58^.
