# Supplementary material for: Site-specific apparent optimum air temperature for vegetation photosynthesis across the globe
Source: Sci Data. 2024 Jul 11;11:758. doi: 10.1038/s41597-024-03603-7 (PMC11239885; doi:10.1038/s41597-024-03603-7)
Supplement: Supplementary file 1 — Supplementary Online materials for “Site-specific apparent optimum air temperature for vegetation photosynthesis across the globe” [file 41597_2024_3603_MOESM1_ESM.docx]

# Supplementary Online materials for “Site-specific apparent optimum air temperature for vegetation photosynthesis across the globe”

Li Pan *et al.*

Corresponding author: [xiangming.xiao@ou.edu](mailto:xiangming.xiao@ou.edu) (X. Xiao), School of Biological Sciences, University of Oklahoma, 101 David L. Boren Blvd., Norman, OK 73019, USA.

**The PDF file includes:**

Supplementary method 1 to 2

Figs. S1 to S5

# Table of Contents

[Supplementary Online materials for Site-specific apparent optimum air temperature for vegetation photosynthesis across the globe 1](#_Toc168050662)

[Supplementary methods 1. Optimum air temperature derived from GPP ($\text{Topt}\text{-}\text{siteGPP}$) 2](#_Toc168050663)

[Supplementary methods 2. Simple linear regression 2](#_Toc168050664)

[Figure S1. The workflow for estimating optimum temperature for vegetation productivity in the southern hemisphere (Amazon Forest, BR-Sa3, EBF, 3.0180°S, 54.9714°W). a. raw LST (LST_raw_) and reconstructed LST (LST_re_) time series. The thermal growing season is defined as the period LST_re_ greater than 5°C (light blue filled area); b. raw EVI (EVI_row_, only after cloud removal), gap filled EVI (EVI_gapfill_, linear interpolation) and smoothed EVI (EVI_smooth_, S-G filter); c. The curve of T_air_ response to EVI. The solid green curve is fitting curves using cubic regression splines. c. also shows a boxplot with points every 2°C bin; d. interannual variations of T_opt-site-year_ from 2000 to 2019, T_opt-site_ is calculated as the median value from T_opt-site-year_. 3](#_Toc168050665)

[Figure S2: Geographical distribution of 14 biome ecosystems in MODIS land cover products (MCD12Q1). This map only shows pixels with constant land cover type from 2000 to 2019. ENF: Evergreen Needleleaf Forests; EBF: Evergreen Broadleaf Forests DNF: Deciduous Needleleaf Forests; DBF: Deciduous Broadleaf Forests; MF: Mixed Forests; CSH: Closed Shrublands; OSH: Open Shrublands; WSA: Woody Savannas; SAV: Savannas; GRA: Grasslands; WET: Permanent Wetlands; CRO: Croplands; URB: Urban and Built-up Lands; CNV: Cropland/Natural Vegetation Mosaics. 4](#_Toc168050666)

[Figure S3. Global map of T_opt-site_ at four period (2000-2004, 2005-2009, 2010-2014, and 2015-2019). Each map is the median value of the T_opt-site-year_ during the period. 5](#_Toc168050667)

[Figure S4. The Seasonal dynamics of NDVI, EVI, NIRv, and T_air-DT_ in 2010 at Harvard Forest site. 6](#_Toc168050668)

[Figure S5: Global maps of optimum air temperature derived from different VIs. a. global map of T_opt-site_ derived from the NDVI-T_air-DT_ response curve; c. global map of T_opt-site_ derived from the NIRv-T_air-DT_ response curve. The global map only shows the area where annual NDVI is larger than 0.1. The histogram represents the proportion of pixels within the different intervals, as shown on the x-axis. The red line represents the Kernel Density Estimation (KDE). b and d correspond to the average values across latitude gradient in and c, respectively. The solid black lines are calculated as the average value for all pixels within 1° of latitude, and the shadow indicates the standard deviation. 7](#_Toc168050669)

# Supplementary methods 1. Optimum air temperature derived from GPP ($\text{T}_{\text{opt}\text{-}\text{site}}^{\text{GPP}}$)

We selected 137 eddy flux tower sites, encompassing 11 out of 14 biomes (DNF, URB, and CNV were excluded due to their very limited number of sites). The eddy flux tower data were acquired from the AmeriFlux data portal (http://ameriflux.ornl.gov/) and the Fluxnet data portal (https://fluxnet.org/). These sites provide Gross Primary Production (GPP) data at half-hourly or hourly intervals. We excluded nighttime GPP data, defining daytime periods as those with Photosynthetic Photon Flux Density (PPFD) greater than 0. Subsequently, GPP data at half-hour or hour intervals were aggregated into daily daytime intervals. To align with the temporal resolution of the EVI and T_air-DT_, we calculated the average values over 8-day periods as the representative daily average. The $\text{T}_{\text{opt}\text{-}\text{site-year}}^{\text{GPP}}$ was derived from the annual response curves of GPP to T_air-DT_. The $\text{T}_{\text{opt}\text{-}\text{site}}^{\text{GPP}}$ is calculated from the median of all $\text{T}_{\text{opt}\text{-}\text{site-year}}^{\text{GPP}}$.

# Supplementary methods 2. Simple linear regression

Simple linear regression is a statistical method that models the relationship between two variables by fitting a linear equation to observed data. Performance and fit are often assessed using metrics of the coefficient of determination (R^2^), indicating the proportion of the variance in the dependent variable predictable from the independent variable. R^2^ without an intercept can be calculated as:

$$\begin{aligned} R^{2}=1-\frac{\sum({\text{y}_{\text{i}}-\hat{y})}^{\text{2}}}{\sum{(\text{y}_{\text{i}}-\bar{y})}^{\text{2}}}\#\left( 1 \right) \end{aligned}$$

Where $\text{y}_{\text{i}}$ is the i-th value of the independent variable y. $\hat{y}$ is the i-th predicted value. $\bar{y}$ is the mean value. We also calculated the Standard Deviation (S.D.), which is a statistic used to measure the dispersion of a set of data The S.D.can be estimated as:

$$\begin{aligned} \text{S.D. }=\sqrt{\frac{1}{\text{N}}\sum\left( \text{A}_{\text{i}}- \text{μ} \right)^{2}}\#\left( 2 \right) \end{aligned}$$

Where N is the number of data, $\text{A}_{\text{i}}$ is the value of each data, and μ is the mean value.


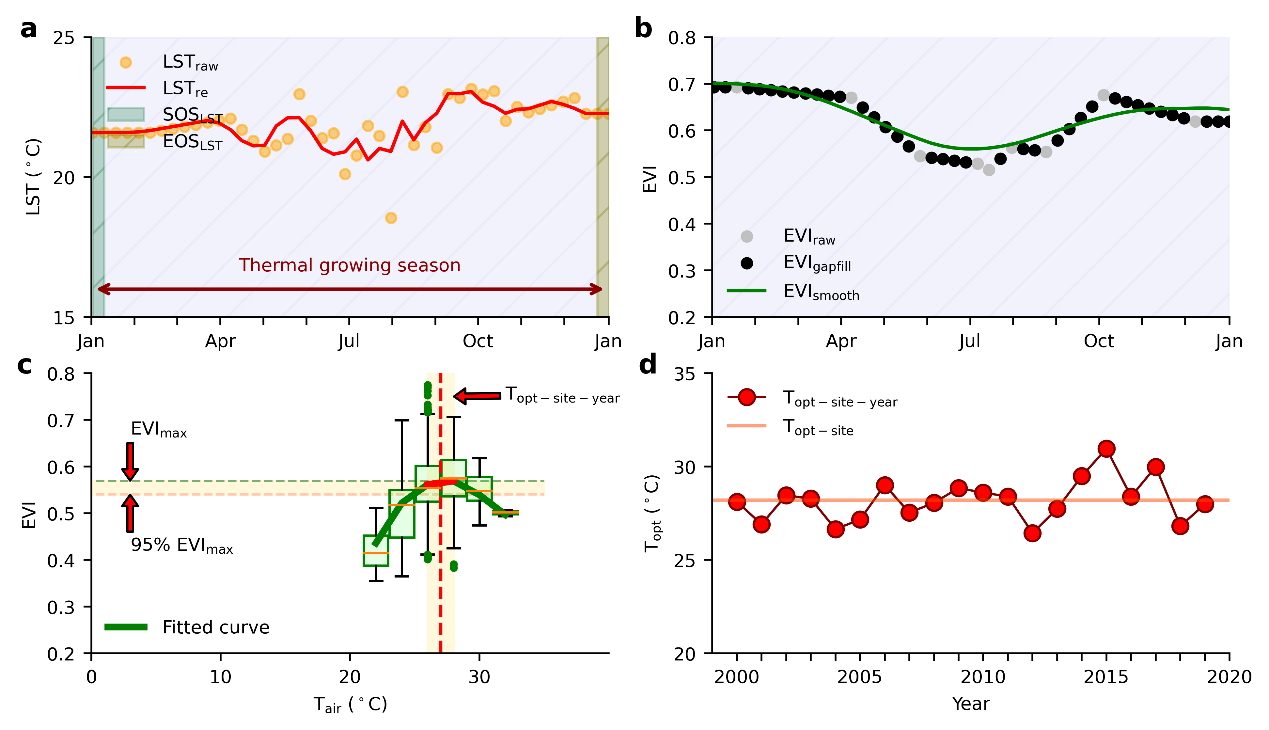


# Figure S1. The workflow for estimating optimum temperature for vegetation productivity in the southern hemisphere (Amazon Forest, BR-Sa3, EBF, 3.0180°S, 54.9714°W). a. raw LST (LST_raw_) and reconstructed LST (LST_re_) time series. The thermal growing season is defined as the period LST_re_ greater than 5°C (light blue filled area); b. raw EVI (EVI_row_, only after cloud removal), gap filled EVI (EVI_gapfill_, linear interpolation) and smoothed EVI (EVI_smooth_, S-G filter); c. The curve of T_air_ response to EVI. The solid green curve is fitting curves using cubic regression splines. c. also shows a boxplot with points every 2°C bin; d. interannual variations of T_opt-site-year_ from 2000 to 2019, T_opt-site_ is calculated as the median value from T_opt-site-year_.


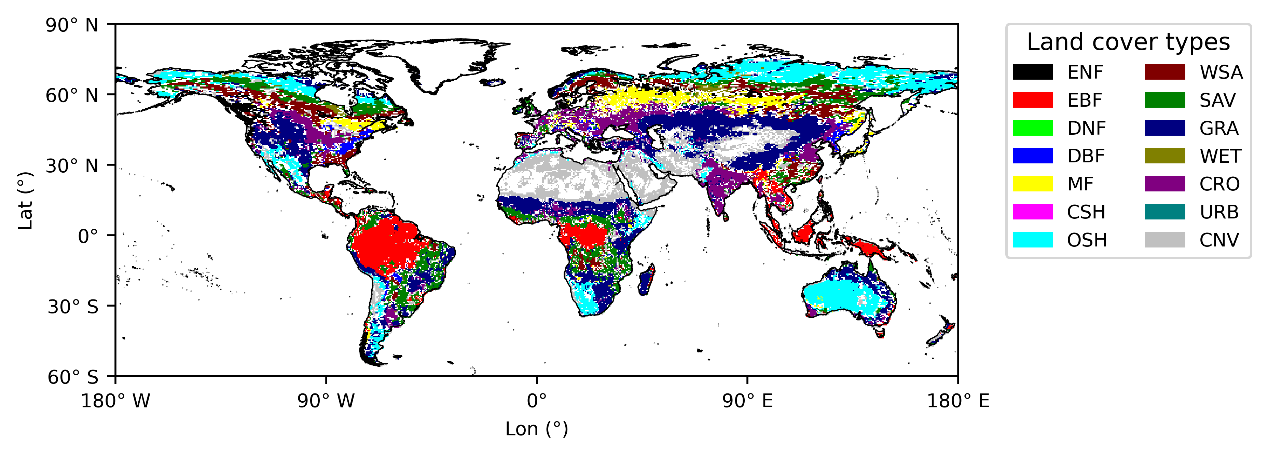


# Figure S2: Geographical distribution of 14 biome ecosystems in MODIS land cover products (MCD12Q1). This map only shows pixels with constant land cover type from 2000 to 2019. ENF: Evergreen Needleleaf Forests; EBF: Evergreen Broadleaf Forests DNF: Deciduous Needleleaf Forests; DBF: Deciduous Broadleaf Forests; MF: Mixed Forests; CSH: Closed Shrublands; OSH: Open Shrublands; WSA: Woody Savannas; SAV: Savannas; GRA: Grasslands; WET: Permanent Wetlands; CRO: Croplands; URB: Urban and Built-up Lands; CNV: Cropland/Natural Vegetation Mosaics.


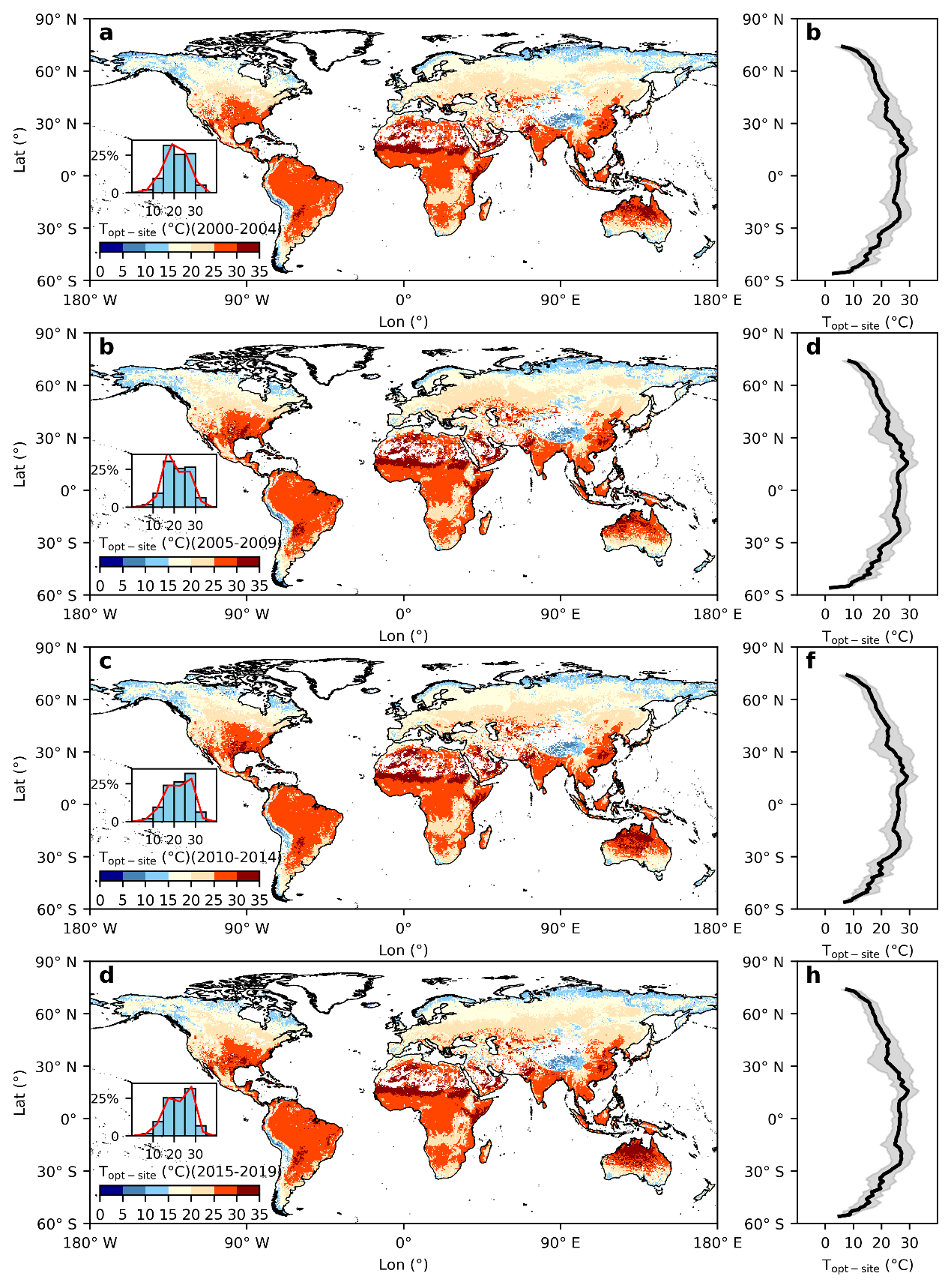


# Figure S3. Global map of T_opt-site_ at four period (2000-2004, 2005-2009, 2010-2014, and 2015-2019). Each map is the median value of the T_opt-site-year_ during the period.


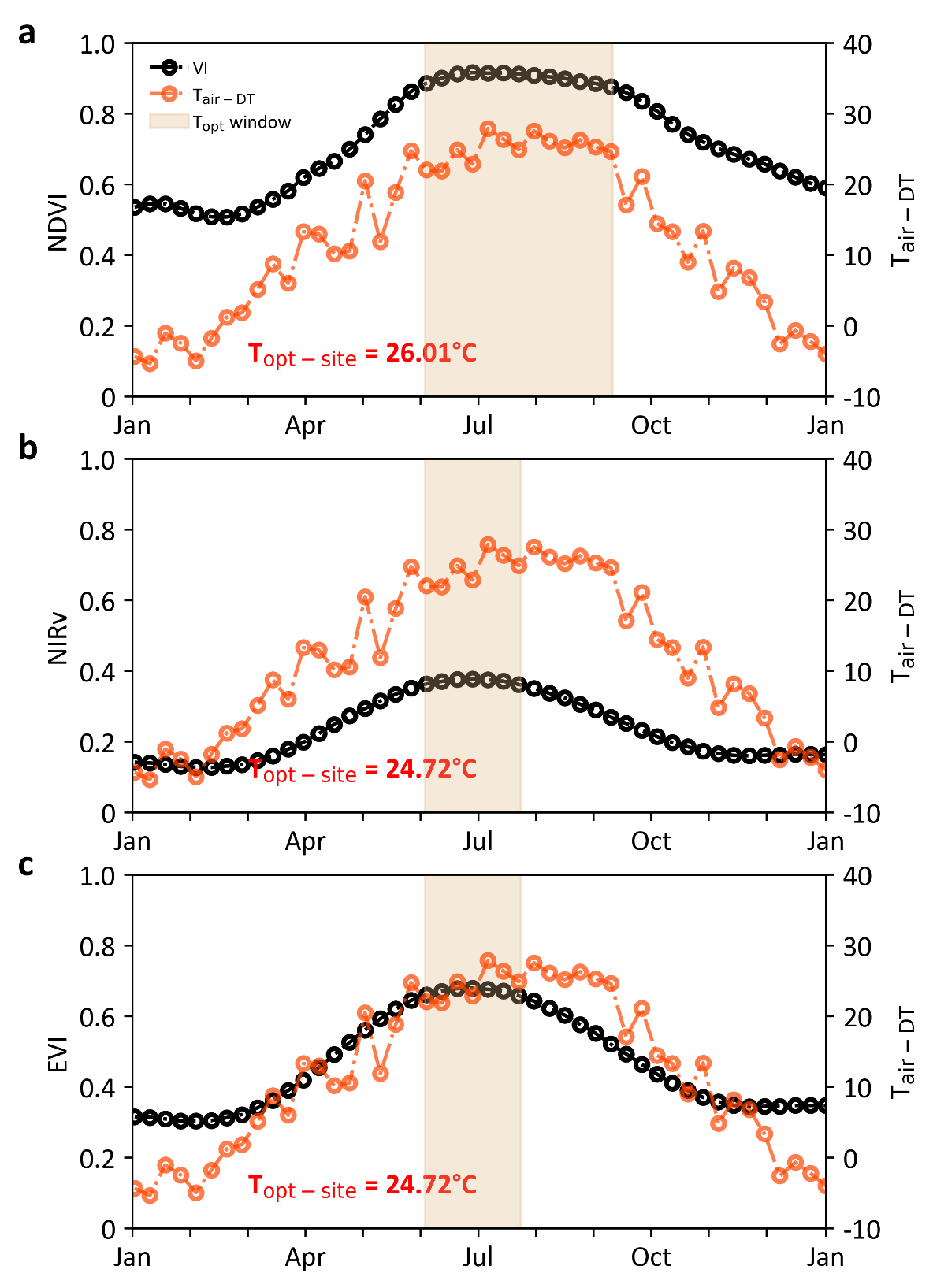


# Figure S4. The Seasonal dynamics of NDVI, NIRv, EVI and T_air-DT_ in 2010 at Harvard Forest site.


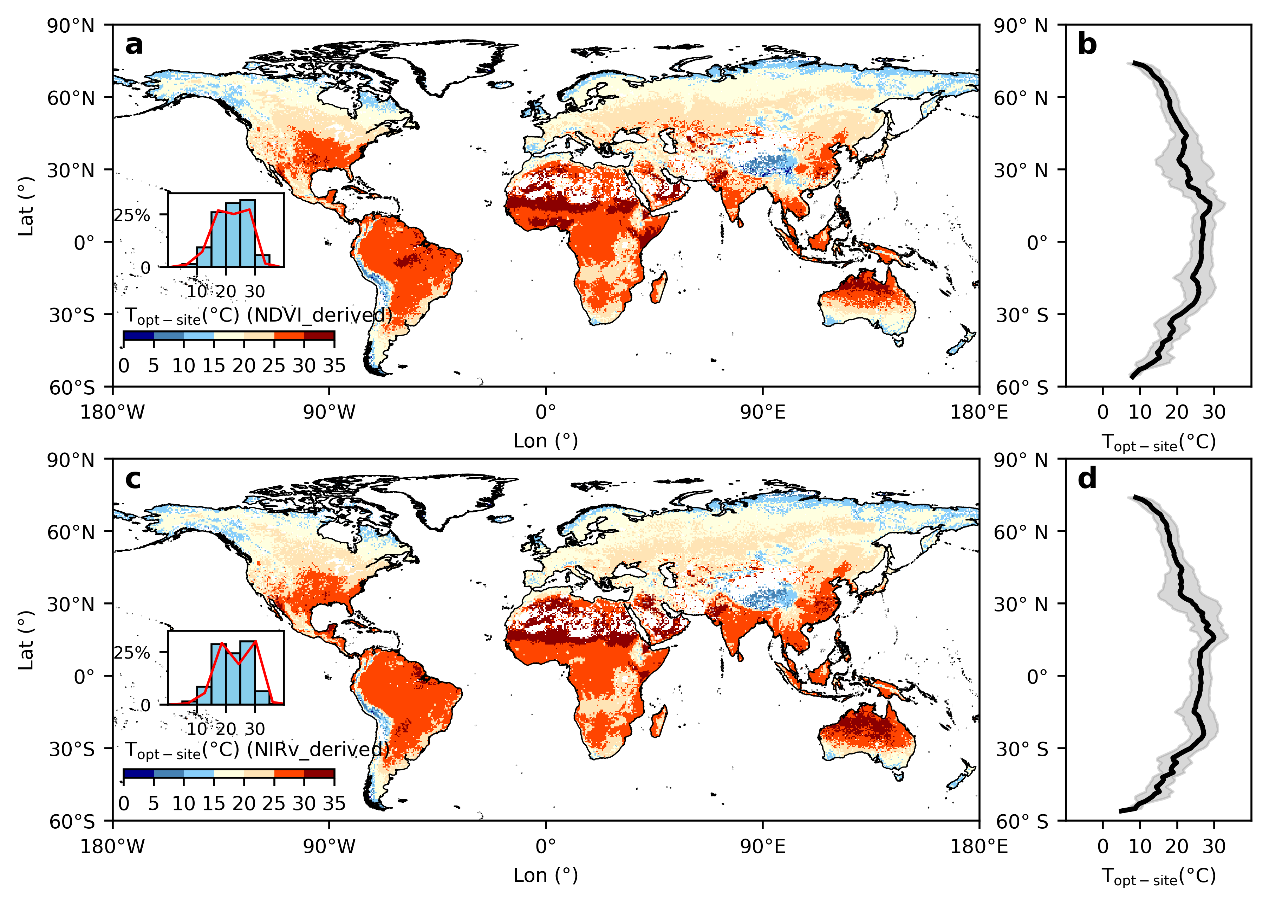


# Figure S5: Global maps of optimum air temperature derived from different VIs. a. global map of T_opt-site_ derived from the NDVI-T_air-DT_ response curve; c. global map of T_opt-site_ derived from the NIRv-T_air-DT_ response curve. The global map only shows the area where annual NDVI is larger than 0.1. The histogram represents the proportion of pixels within the different intervals, as shown on the x-axis. The red line represents the Kernel Density Estimation (KDE). b and d correspond to the average values across latitude gradient in and c, respectively. The solid black lines are calculated as the average value for all pixels within 1° of latitude, and the shadow indicates the standard deviation.
